# Real-World Assessment of Recommended COVID-19 Vaccination Waiting Period after Chemotherapy

**DOI:** 10.3390/vaccines12060678

**Published:** 2024-06-18

**Authors:** Kai-Wen Cheng, Chi-Hua Yen, Renin Chang, James Cheng-Chung Wei, Shiow-Ing Wang

**Affiliations:** 1 Department of Emergency Medicine, China Medical University Hospital, Taichung 40447, Taiwan; kevinman@ms6.hinet.net; 2 School of Medicine, Chung Shan Medical University, Taichung 40201, Taiwan; chaseyen@gmail.com; 3 Department of Family and Community Medicine, Chung Shan Medical University Hospital, Taichung 40201, Taiwan; 4 Department of Medical Education and Research, Kaohsiung Veterans General Hospital, Kaohsiung 81362, Taiwan; rhapsody1881@gmail.com; 5 Department of Recreation and Sports Management, Tajen University, Pintung 90741, Taiwan; 6 Institute of Medicine, Chung Shan Medical University, Taichung 40201, Taiwan; 7 Department of Nursing, Chung Shan Medical University, Taichung 40201, Taiwan; 8 Department of Allergy, Immunology & Rheumatology, Chung Shan Medical University Hospital, Taichung 40201, Taiwan; 9 Graduate Institute of Integrated Medicine, China Medical University, Taichung 40447, Taiwan; 10 Office of Research and Development, Asia University, Taichung 41354, Taiwan; 11 Center for Health Data Science, Department of Medical Research, Chung Shan Medical University Hospital, Taichung 40201, Taiwan

**Keywords:** COVID-19, vaccination, chemotherapy, cancer patient, immunology

## Abstract

There is a knowledge gap concerning the proper timing for COVID-19 vaccination in cancer patients undergoing chemotherapy. We aimed to evaluate the suitability of the guidelines that recommend waiting at least three months after undergoing chemotherapy before receiving a COVID-19 vaccine. This retrospective cohort study used aggregated data from the TriNetX US Collaboratory network. Participants were grouped into two groups based on the interval between chemotherapy and vaccination. The primary outcome assessed was infection risks, including COVID-19; skin, intra-abdominal, and urinary tract infections; pneumonia; and sepsis. Secondary measures included healthcare utilization and all causes of mortality. Kaplan–Meier analysis and the Cox proportional hazard model were used to calculate the cumulative incidence and hazard ratio (HR) and 95% confidence intervals for the outcomes. The proportional hazard assumption was tested with the generalized Schoenfeld approach. Four subgroup analyses (cancer type, vaccine brand, sex, age) were conducted. Sensitivity analyses were performed to account for competing risks and explore three distinct time intervals. Patients receiving a vaccine within three months after chemotherapy had a higher risk of COVID-19 infection (HR: 1.428, 95% CI: 1.035–1.970), urinary tract infection (HR: 1.477, 95% CI: 1.083–2.014), and sepsis (HR: 1.854, 95% CI: 1.091–3.152) compared to those who adhered to the recommendations. Hospital inpatient service utilization risk was also significantly elevated for the within three months group (HR: 1.692, 95% CI: 1.354–2.115). Adhering to a three-month post-chemotherapy waiting period reduces infection and healthcare utilization risks for cancer patients receiving a COVID-19 vaccine.

## 1. Introduction

The coronavirus 2019 (COVID-19) pandemic has been considered the most severe health and economic crisis of the century [1]. According to the World Health Organization (WHO), there were 770,778,396 confirmed cases of COVID-19 and 6,958,499 deaths worldwide as of 21 September 2023 [2].

Cancer patients face an increased risk of a severe COVID-19 infection and related complications, emphasizing the importance of vaccination [3,4,5,6]. However, their exclusion from vaccine clinical trials has raised safety and efficacy concerns [7], leading to lower vaccine acceptance rates [8], especially among those receiving chemotherapy.

Chemotherapy is a modality of cancer therapies that involves the administration of chemical agents to destroy cancer cells by interfering with macromolecular synthesis and function, including DNA, RNA, and protein synthesis, or by disrupting the proper functioning of preformed molecules [9,10]. However, chemotherapy targets fast-growing cells and cannot distinguish between cancer and normal cells. This leads to chemotherapeutic drugs causing significant immunosuppressive side effects, either by directly inhibiting or killing immune effector cells or indirectly by inducing anergy or immune paralysis [9,10,11].

The Centers for Disease Control and Prevention (CDC) and the National Comprehensive Cancer Network (NCCN) recommend waiting at least three months after undergoing chemotherapy before receiving a COVID-19 vaccine, based on data from other vaccines indicating that they tend to have reduced effectiveness when administered when patients are immunosuppressed [12,13,14]. Ethical concerns precluded the enrollment of cancer patients in COVID-19 vaccine evaluations; therefore, a knowledge gap regarding the appropriate time from chemotherapy to COVID-19 vaccination exists.

This study aimed to use real-world data to validate the suggested three-month waiting period, helping healthcare professionals and patients make informed decisions about balancing the risk of COVID-19 against potential vaccination-related risks during the immunosuppressed period.

## 2. Materials and Methods

### 2.1. Study Design and Data Source

This retrospective cohort analysis, conducted in September 2023, utilized data from 1 January 2020 to 31 December 2022, sourced from TriNetX, a comprehensive real-world data ecosystem in healthcare and life sciences. TriNetX aggregates de-identified electronic health records from more than 250 million individuals [15]. Data quality in TriNetX is ensured through standardized metrics, such as conformance, completeness, and plausibility [16], and it has been employed in various high-quality studies [17,18].

### 2.2. Study Subjects

Eligible cancer patients were identified using International Statistical Classification of Diseases, Tenth Revision, Clinical Modification (ICD-10-CM) codes (C00-C96). Patients under 18 or those not receiving their first dose of COVID-19 vaccine, as indicated by CPT codes 0001A, 0011A, 0021A, and 0041A (details in Supplementary Table S1), were excluded.

The participants were divided into two groups based on the interval between chemotherapy (TriNetX-curated code 1002) and vaccination: one group received a COVID-19 vaccine within three months after chemotherapy, and the other received it three months or more after chemotherapy. The index date for the groups was set to the date of the first dose of COVID-19 vaccine. Patients were excluded if they experienced outcomes of interest, received critical care services or admitted hospitalization, or died within three months of or before the index date. The groups were followed for 30 days after the index date to assess short-term responses.

### 2.3. Outcomes

The risk of infections was examined in cancer patients, including COVID-19 infection (defined by ICD-10 codes U07, U09, B34.2, J12.81, J12.82, Z86.16, or positive COVID-19 RNA test results), pneumonia (J12–J18), skin infection (L02–L03, L08), intra-abdominal infection (A09, K83.0, K67), urinary tract infection (N39.0), and severe infection (sepsis, A41, R65.2) were examined. Medical utilization incidences, including hospitalization, critical care services, and all causes of mortality, were analyzed as secondary outcomes.

### 2.4. Covariates

To mitigate confounding effects, various baseline covariate factors identified one year before the index date were considered in this study. Demographic variables included age at the index date, sex, race, and socioeconomic status (using ICD-10 code Z59 as a proxy for housing and economic circumstances). Lifestyle variables encompassed tobacco use (ICD-10 code Z72.0, serving as a proxy for smoking), nicotine dependence (F17), and alcohol-related disorders (F10, serving as a proxy for alcohol consumption). Medical utilization comprised office or other outpatient services (CPT code 1013626), emergency department services (1013711), hospital inpatient services (1013659), and preventive medicine services (1013829).

Information on cancer sites was included, categorized into 16 groups based on anatomical locations and identified using ICD-10 codes.

Comorbidities were classified as present or absent, defined using ICD-10 codes, such as essential hypertension (I10), overweight and obesity (E66), hyperlipidemia, unspecified (E78.5), vitamin D deficiency (E55), diabetes mellitus (08-E13), asthma (J45), other chronic obstructive pulmonary diseases (J44), liver diseases (K70-K77), diseases of the blood and blood-forming organs and certain disorders involving the immune mechanism (D50-D89), depressive episode (F32), anxiety, dissociative, stress-related, somatoform, and other nonpsychotic mental disorders (F40-F48), and sleep disorders (G47).

Laboratory results, including the White Blood Cell/Leukocyte count and the differential count ratios (lymphocytes, monocytes, eosinophils, basophils, neutrophils), were also incorporated into the analysis.

### 2.5. Statistical Analyses

To mitigate the influence of confounding factors, we employed TriNetX’s built-in capability to generate a 1:1 propensity score matching (PSM) using greedy nearest-neighbor matching [19]. A caliper pooled standard deviation of 0.1 was utilized for all listed characteristics during the matching process. Standardized mean differences (SMDs) were used to assess the balance of characteristics between the two groups, with SMD values below 0.1 indicating that the groups were well matched.

Kaplan–Meier analysis and the Cox proportional hazard model were used to calculate the cumulative incidence and hazard ratio (HR) with 95% confidence intervals for the outcomes. The proportional hazard assumption was tested with the generalized Schoenfeld approach, computed using R’s Survival package v3.2-3 [20]. Log-rank tests within the TriNetX platform determined differences in the survival curves.

Subgroup analyses were conducted to examine the differences between the groups based on cancer type (solid organ cancers/lymphoid, hematopoietic, and related tissue cancers), vaccine (BNT162b2/mRNA-1273), and sex (male/female). Considering that the immunity level seems age-related [21,22], we also presented the baseline characteristics and subgroup analysis for those aged 18–64 and those aged 65 and above.

**Figure 1:**
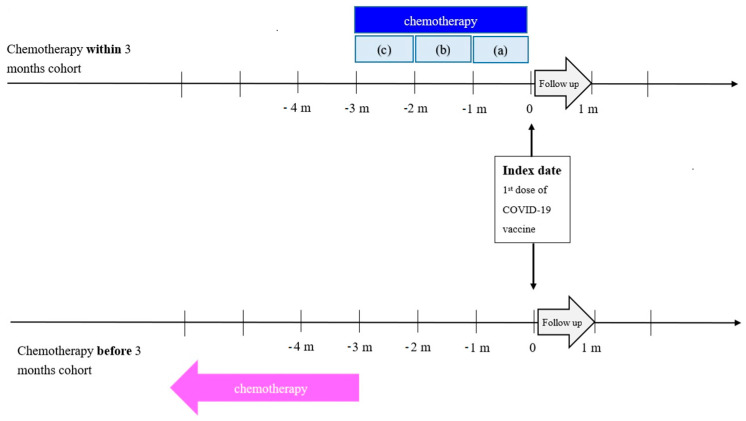
Study design.

Two sensitivity analyses were performed to demonstrate the consistency of our results. It is important to account for the potential influence of competing risks when one or more outcomes that could potentially interfere with the primary outcome are encountered, particularly in the case of cancer patients. Therefore, using the solution proposed by Manja et al. [23], we incorporated the competing event (death) into each endpoint. In addition, the patients vaccinated within three months after chemotherapy were divided into three distinct time intervals: within one month, within one to two months, and within two to three months after chemotherapy. We subsequently conducted separate comparisons between patients from these time intervals and those who received the vaccine more than three months after chemotherapy. Figure 1 illustrates the definitions of the two cohorts and provides visual representations of these three separate time intervals.

## 3. Results

### 3.1. Characteristics of Study Subjects

**Figure 2:**
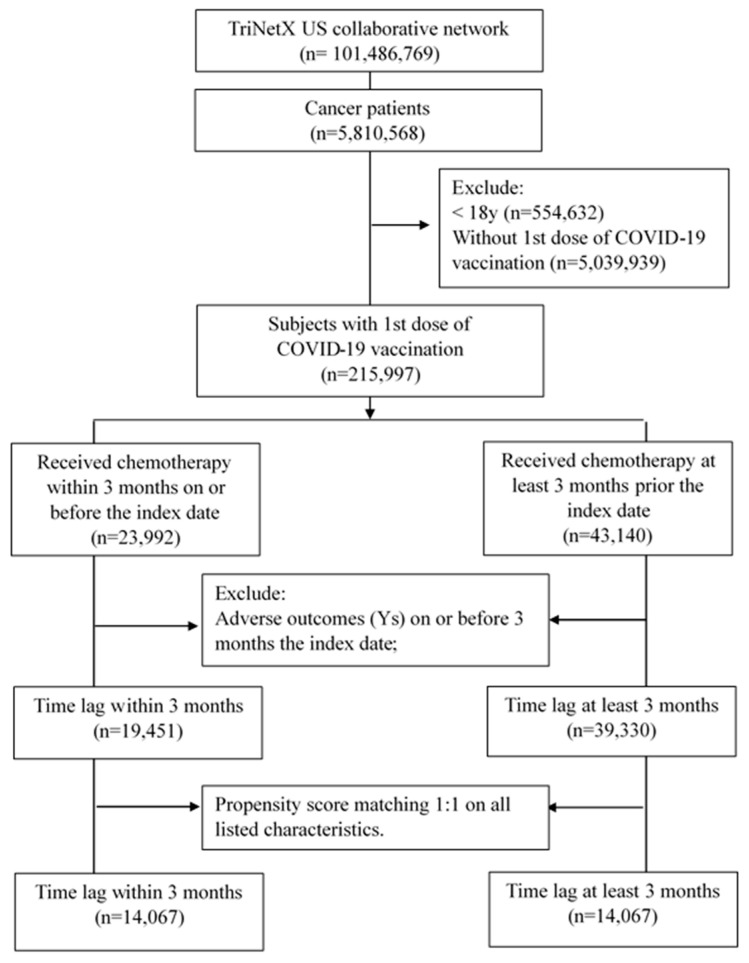
Selection process.

After propensity score matching, we identified a total of 14,067 patients who had received COVID-19 vaccination within 3 months after chemotherapy, while an equal number delayed vaccination beyond 3 months. Figure 2 illustrates the selection process. The subjects’ baseline characteristics are presented in Supplementary Table S2. Prior to matching, differences in age at the index date, cancer site, comorbidities, and laboratory data existed between the two groups. However, after matching, these differences were within an acceptable range (SMD < 0.1). Even when stratified into different age groups to reveal deeper insights, the baseline characteristics of both groups still demonstrated comparable distribution patterns. Please refer to Table S3 for individuals aged 18–64 years and Table S4 for those aged 65 years and older.

### 3.2. Outcomes

**Figure 3:**
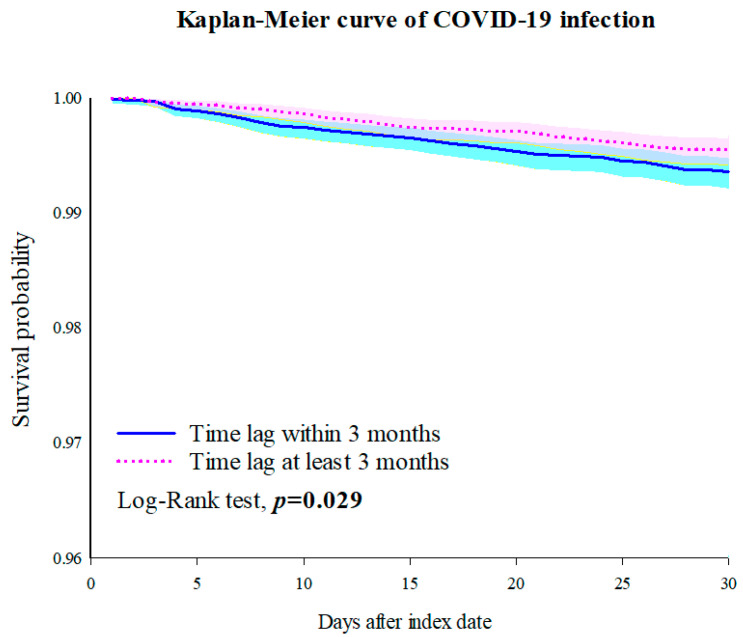
KM curve of COVID-19 infection.

Table 1 displays the number of patients with each outcome, along with the 30-day adjusted HR for the incidence of infections. Patients who received a vaccine within three months showed elevated risks of COVID-19 infections (HR: 1.428, 95% CI: 1.035–1.970), urinary tract infections (HR: 1.477, 95% CI: 1.083–2.014), and sepsis (HR: 1.854, 95% CI: 1.091–3.152) compared to those who received a vaccine at least three months after chemotherapy (Figure 3, Supplementary Figures S1 and S2). Similar results were obtained after adjusting for various variables (Supplementary Table S5).

Patients who received a vaccine within three months had a significantly higher risk of hospital admission (HR: 1.692, 95% CI: 1.354–2.115) than those who received a vaccine more than three months after chemotherapy (Supplementary Figure S3). There was no significant difference in all-cause mortality between the two groups (HR: 1.995, 95% CI: 0.998–3.989).

### 3.3. Subgroup Analyses

Among patients with solid organ cancers, those who received a vaccine within three months had significantly higher risks of using hospital inpatient services (HR: 1.784, 95% CI: 1.392–2.288) and critical care services (HR: 2.197, 95% CI: 1.193–4.045) than those who received a vaccine at least three months after chemotherapy (Supplementary Table S6). Conversely, individuals with lymphoid, hematopoietic, and related tissue cancers exhibited no significant differences in outcomes between the two groups.

The patients who received the BNT162b2 vaccine within three months after chemotherapy had significantly higher risks of urinary tract infections (HR: 1.717, 95% CI: 1.157–2.548) and increased hospital admission (HR: 1.862, 95% CI: 1.416–2.447) than those who received BNT162b2 vaccine at least three months post-chemotherapy. The patients who received the mRNA-1273 vaccine within three months had slightly elevated risks of COVID-19 infections, urinary tract infections, and hospitalizations; however, these differences were not statistically significant (Supplementary Table S7).

For male patients, no significant outcome differences were observed between the two groups (Supplementary Table S8). However, female patients who had a vaccine within three months had a significantly higher risk of hospital admission (HR: 1.819, 95% CI: 1.331–2.486) than those who received a vaccine at least three months after chemotherapy.

In the 18–64 years age group, the patients vaccinated within three months had elevated risks of COVID-19 infections (HR: 1.607, 95% CI: 1.002–2.575), urinary tract infections (HR: 2.697, 95% CI: 1.305–5.571), and increased hospital admission (HR: 1.800, 95% CI: 1.191–2.722) than those who received a vaccine at least three months after chemotherapy (Supplementary Table S9). Among the patients over 64 vaccinated within three months, there was a significantly higher risk of hospital admission (HR: 1.812, 95% CI: 1.376–2.384) than those who received a vaccine more than three months after chemotherapy.

### 3.4. Sensitivity Analyses

In the sensitivity analysis accounting for competing risk, patients vaccinated within three months had significantly increased risks of sepsis (HR: 1.552, 95% CI: 1.021–2.359) and utilization of hospital inpatient services (HR: 1.622, 95% CI: 1.309–2.010) and critical care services (HR: 1.485, 95% CI: 1.000–2.206) than those who received a vaccine after more than three months (Supplementary Table S10).

Patients who were vaccinated within one month and between one and two months after chemotherapy had significantly higher risks of urinary tract infections and sepsis (HR: 2.026 and 1.930, respectively), and hospital admission (HR: 1.923 and 2.279, respectively) compared to their matched counterparts who were vaccinated at least three months after chemotherapy (Table 2).

## 4. Discussion

This retrospective cohort study using the TriNetX US network found higher risks of infections (COVID-19 infection, urinary tract infection, and sepsis) in patients who received a COVID-19 vaccination within three months after chemotherapy compared to those adhering to the recommended guideline of waiting at least three months after chemotherapy.

Previous studies have emphasized that the substantial and enduring effects of chemotherapy are most pronounced in terms of the levels of circulating T-cells and B-cells and the development of neutropenia [24,25]. These impacts can compromise a patient’s immune system for weeks to several months or even longer after chemotherapy [26,27]. Immune recovery depends on the intricate interplay between bone-marrow-derived T-cell progenitors and the nurturing thymic stromal microenvironment [28]. Generally, neutrophil and monocyte counts return to normal early, while B-cells take about three months to recover [26,29,30,31]. Therefore, many guidelines or expert groups recommend that cancer patients wait at least three months after chemotherapy for COVID-19 vaccination [12,13,32,33]. However, it is important to note that this timeline is based on studies of healthy individuals and may not necessarily apply to cancer patients whose immune systems may be compromised following chemotherapy. Our findings align with the guideline recommendation, indicating that waiting at least three months after chemotherapy before COVID-19 vaccination offers greater benefits than receiving the vaccination within three months.

Furthermore, our sensitivity analysis demonstrated that a shorter interval between chemotherapy and vaccine is associated with a more pronounced risk of severe infections, such as sepsis and UTIs, and hospitalization. Velardi et al. previously investigated T-cell regeneration in patients who had undergone hematopoietic cell transplantation, revealing a consistent pattern of immune cell reconstitution, in contrast to the rapid recovery observed in innate immune cells, such as neutrophils, natural killer (NK) cells, and monocytes; adaptive immune cells, specifically T-cells, demonstrated a notably slower recovery rate and increased susceptibility to adverse effects stemming from infections or therapeutic interventions, such as cytoreductive chemotherapy or radiotherapy [28]. CD8^+^ T-cells and B-cells typically show initial recovery at around 30 days, peaking at about 100 days. The results of this study, to a certain degree, also corroborate this time frame.

This study revealed that among individuals with lymphoid, hematopoietic, and related tissue cancers, the period between chemotherapy and vaccination does not significantly influence the subsequent risk of infections. A possible reason for this is that the vaccine protection levels are generally higher for patients with solid cancers than those with hematologic malignancies [34,35,36,37,38]. Previous reports have indicated that hematologic cancers often involve B-cell defects, leading to reduced rates (46–85%) of antibody response or seroconversion to vaccines [39]. Several studies have observed vaccine ineffectiveness or failure in immunosuppressed patients [37,40,41,42,43,44]. Additionally, hematological malignancies encompass myeloid and lymphatic tumors that disrupt regular hematopoietic function, posing a dual challenge for patients due to the close relationship between immune reconstitution and hematopoietic recovery.

This study revealed that the timing of vaccination after chemotherapy had a more significant impact on infection risks among patients receiving the BNT162b2 vaccine, with less variation observed in those receiving the mRNA-1273 vaccine. Despite both vaccines utilizing mRNA technology, there are dosage differences. The BNT162b2 vaccine delivers 30 µg of mRNA that encodes full-length SARS-CoV-2 Spike protein per dose along with other ingredients, whereas the mRNA-1273 vaccine contains 100 µg SARS-CoV-2 mRNA per dose. Research suggests that the mRNA-1273 vaccine elicits a more robust immune response, characterized by higher levels of neutralizing antibodies [45,46,47,48], fewer breakthrough infections [49,50,51], and longer-lasting vaccine efficacy [48,52]. It is also possible that the higher dosage of the mRNA-1273 vaccine may contribute to enhanced immune response and consequently lead to increased protection.

There was also an elevated risk of infections in patients who received the COVID-19 vaccine within three months after chemotherapy, particularly in younger individuals. This observation aligns with the notion that thymic T-cell production significantly declines with age, with a reduction ranging from 10- to 100-fold. Immune competence diminishes with age, and there is a decreased abundance of CD4^+^ and CD8^+^ T-cells, a reduced compartment size, and increased clonality [53]. Furthermore, previous studies have indicated an inverse correlation between age and neutralizing responses after vaccination, indicating that vaccine responses are compromised in older individuals. Potential reasons for the diminished neutralizing responses may encompass the reduced antibody concentrations (quantity) or antibodies with lower affinity (quality) arising from B-cell selection, decreased CD4^+^ T-cell assistance, or a combination of these factors [54,55].

This study possesses several notable strengths. First, this study involved a thorough evaluation of the guideline recommendations using real-world data to compensate for the lack of relevant information in practical scenarios. Second, this study focused exclusively on cancer patients who had received their first COVID-19 vaccine, enhancing comparability and reducing selection bias. Furthermore, this study presents four models that adjust for various variables and effectively capture diverse real-world scenarios. Considering the importance of socioeconomic status and lifestyle habits in influencing outcomes, we opted to substitute proxy variables in their place and included them as part of the study variables. Additionally, we conducted multiple subgroup analyses based on cancer type, vaccine brand, sex, and age to assess the applicability of the recommendation to diverse subpopulations. Sensitivity analyses accounting for competing risks were carried out. Moreover, we conducted a more granular analysis by dividing the patients vaccinated within three months into three distinct timeframes, attempting to clarify the correlation between the time lag and subsequent infection. These aspects contributed to providing precise clinical recommendations based on our research findings.

However, this study has several limitations. The present study is a retrospective study, which can only consider limited variables and is easily influenced by various factors, introducing confounding bias. Therefore, we cannot definitively ascertain whether the outcome risk is attributable to the vaccine or to preceding chemotherapy [56,57]. The proper time interval between chemotherapy and vaccination can vary depending on factors including the type and intensity of chemotherapy, the specific chemotherapy drugs, the patient’s underlying conditions, and cancer severity. Additionally, it was unclear from the analysis whether people had completed chemotherapy or were still receiving it at the time of their vaccinations. The absence of these factors in our study considerations could potentially introduce confounding bias. Patients who were not vaccinated within 3 months of chemotherapy had to survive to that time point to be included in the analysis, which may introduce immortal time bias that is difficult to reconcile. Although we have made every effort to balance the baseline conditions of the two groups (especially regarding the level of immunosuppression and immune reconstitution), we were unable to assess individual responses to vaccination. Future studies can delve deeper to overcome this limitation. In addition, the database we employed is not derived from comprehensive population-wide health records, which may result in a loss of follow-ups or patients seeking care at HCOs outside the TriNetX network. This situation has the potential to introduce bias into the findings and also restricts the generalizability of the study results. The absence of cause-of-death information in the electronic medical record system hinders the ability to devise potential intervention measures to prevent mortality. The use of proxy variables for socioeconomic status and lifestyle could introduce bias. Moreover, due to the constraints in the platform’s analytical capabilities, our analysis was restricted to patients who had received their first vaccine dose. Future research should explore the effects of different vaccine doses or boosters to better represent real-world scenarios.

## 5. Conclusions

Our study underscores the importance of cancer patients adhering to the guideline of waiting at least three months after chemotherapy before receiving a COVID-19 vaccine. This practice significantly lowers the risk of infections and adverse clinical outcomes, particularly in patients with solid organ cancers and those aged 18–64. Our findings serve as a valuable reference for clinical physicians and healthcare professionals in providing appropriate care for cancer patients.

The CDC and NACC guidelines are based on data from other vaccines. However, due to ethical considerations and time constraints, it was not feasible to conduct RCTs specifically for cancer patients to validate these guidelines. This study is the first large-scale real-world investigation to assess the appropriateness of these guidelines. Nevertheless, future validation using independent databases or RCTs is necessary. Additionally, if faced with other pandemics, health policymakers should promptly utilize real-world data to provide relevant evidence, aiding clinical physicians and healthcare professionals in their decision-making processes and improving patient outcomes.

## Figures and Tables

**Table 1:** Risk of outcomes (1 day to 30 days).

Outcomes	Patients with Outcome		Adjusted ^a^ Hazard Ratio (95% CI)
	Time Lag within 3 Months (*n* = 14,067)	Time Lag at Least 3 Months (*n* = 14,067)	
Infections			
COVID-19 infection	90	63	1.428 (1.035–1.970)
Pneumonia	59	50	1.178 (0.808–1.717)
Skin infection	52	54	0.961 (0.657–1.406)
Intra-abdominal infection	10	10	0.249 (0.028–2.231)
Urinary tract infection	99	67	1.477 (1.083–2.014)
Severe infection (sepsis)	39	21	1.854 (1.091–3.152)
Medical utilization			
Hospital inpatient services	208	123	1.692 (1.354–2.115)
Critical care services	39	29	1.343 (0.831–2.172)
All-cause mortality			
Deceased	24	12	1.995 (0.998–3.989)

Note: CI: Confidence interval. COVID-19: Coronavirus disease 2019. If the patient’s value is less than or equal to 10, the results show the count as 10. ^a^ Propensity score matching was performed on all listed characteristics.

**Table 2:** Risk of outcomes (1 day to 30 days) stratified by time lag interval (between vaccination and chemotherapy).

Outcomes	Adjusted ^a^ Hazard Ratio (95% CI)			
	(a) −1 Day~ −1 Month vs. at Least 3 Months (*n* = 9053 vs. 9053)	(b) −1 ~ −2 Month vs. at Least 3 Months (*n* = 3708 vs. 3708)	(c) −2 ~ −3 Month vs. at Least 3 Months (*n* = 2752 vs. 2752)	(Origin) −1 Day~ −3 Month vs. at Least 3 Months (*n* = 14,067 vs. 14,067)
Infections				
COVID-19 infection	1.300 (0.874–1.935)	0.726 (0.381–1.382)	1.135 (0.567–2.274)	1.428 (1.035–1.970)
Pneumonia	1.529 (0.979–2.387)	1.999 (0.856–4.672)	0.444 (0.137–1.442)	1.178 (0.808–1.717)
Skin infection	1.144 (0.722–1.812)	0.999 (0.433–2.303)	2.003 (0.752–5.336)	0.961 (0.657–1.406)
Intra-abdominal infection	0.747 (0.167–3.339)	1.000 (0.063–15.99)	1.001 (0.063–15.99)	0.249 (0.028–2.231)
Urinary tract infection	2.026 (1.367–3.004)	1.930 (1.012–3.680)	0.939 (0.464–1.899)	1.477 (1.083–2.014)
Severe infection (sepsis)	2.162 (1.091–4.285)	4.000 (0.849–18.83)	2.252 (0.694–7.314)	1.854 (1.091–3.152)
Medical utilization				
Hospital inpatient services	1.923 (1.457–2.537)	2.279 (1.380–3.763)	1.431 (0.819–2.499)	1.692 (1.354–2.115)
Critical care services	1.820 (1.008–3.289)	0.999 (0.351–2.849)	4.006 (0.851–18.86)	1.343 (0.831–2.172)
All-cause mortality				
Deceased	1.282 (0.637–2.577)	1.747 (0.511–5.968)	3.004 (0.606–14.88)	1.995 (0.998–3.989)

Note: CI: Confidence interval. NA: Not available. ^a^ Propensity score matching was performed on all listed characteristics.

## Data Availability

As an HCO member of TriNetX, Chung Shan Medical University Hospital (CSMUH) has access to the de-identified data in TriNetX. All data related to this study have been presented in the article. The data that support the findings of this study are available from the TriNetX Analytics Network: https://live.trinetx.com/tnx/study/170907/analytics/650a9120adf1fe372010a33e/outcomes/results (accessed on 26 April 2024).

## Supplementary Materials

The following supporting information can be downloaded at: https://www.mdpi.com/article/10.3390/vaccines12060678/s1, Table S1: First dose of COVID-19 vaccine codes; Table S2: Baseline characteristics of study subjects (before and after PSM matching); Table S3: Baseline characteristics of study subjects (18~64 years old); Table S4: Baseline characteristics of study subjects (≥65 years old); Table S5: Risk of outcomes (1 day to 30 days)—4 models; Figure S1: Kaplan–Meier curve of urinary tract infection; Figure S2: Kaplan–Meier curve of severe infection (sepsis); Figure S3: Kaplan–Meier curve of hospitalization; Table S6: Risk of outcomes (1 day to 30 days) stratified by cancer type; Table S7: Risk of outcomes (1 day to 30 days) stratified by vaccine brand; Table S8: Risk of outcomes (1 day to 30 days) stratified by sex; Table S9: Risk of outcomes (1 day to 30 days) stratified by age at index; Table S10: Risk of outcomes (1 day to 30 days) dealing with competing risk (deceased).
