# Supplementary material for: Real-World Assessment of Recommended COVID-19 Vaccination Waiting Period after Chemotherapy
Source: Vaccines (Basel). 2024 Jun 18;12(6):678. doi: 10.3390/vaccines12060678 (PMC11209144; doi:10.3390/vaccines12060678)
Supplement: Supplementary file 1 [file vaccines-12-00678-s001.zip › vaccines-3007714-supplementary.pdf]

## Supplementary Materials

**Table S1.** 1st dose of COVID-19 vaccine codes.

| Code | Description |
|---|---|
| UMLS:CPT:0001A | Immunization administration by intramuscular injection of severe acute respiratory syndrome coronavirus 2 (SARS-CoV-2) (coronavirus disease [COVID-19]) vaccine, mRNA-LNP, spike protein, preservative free, 30 mcg/0.3 mL dosage, diluent reconstituted; first dose ***BNT162b2*** |
| UMLS:CPT:0011A | Immunization administration by intramuscular injection of severe acute respiratory syndrome coronavirus 2 (SARS-CoV-2) (coronavirus disease [COVID-19]) vaccine, mRNA-LNP, spike protein, preservative free, 100 mcg/0.5 mL dosage; first dose ***mRNA-1273*** |
| UMLS:CPT:0021A | Immunization administration by intramuscular injection of severe acute respiratory syndrome coronavirus 2 (SARS-CoV-2) (coronavirus disease [COVID-19]) vaccine, DNA, spike protein, chimpanzee adenovirus Oxford 1 (ChAdOx1) vector, preservative free, 5x1010 viral particles/0.5 mL dosage; first dose ***ChAdOx1-S*** |
| UMLS:CPT:0041A | Immunization administration by intramuscular injection of severe acute respiratory syndrome coronavirus 2 (SARS-CoV-2) (coronavirus disease [COVID-19]) vaccine, recombinant spike protein nanoparticle, saponin-based adjuvant, preservative free, 5 mcg/0.5 mL dosage; first dose ***NVX-CoV2373*** |

Note:
UMLS: Unified Medical Language System
CPT: Current procedural terminology

**Table S2.** Baseline characteristics of study subjects (before and after PSM matching) .

| Variables | Before PSM | | | After PSM [a] | | |
|---|---|---|---|---|---|---|
| | Time lag within 3 months (n=19,451) | Time lag at least 3 months (n=39,330) | SMD | Time lag within 3 months (n=14,067) | Time lag at least 3 months (n=14,067) | SMD |
| **Age at index, y** | | | | | | |
| Mean ± SD | 65.3 ± 12.6 | 66.9 ± 12.6 | **0.127** | 66.0 ± 12.3 | 65.8 ± 12.9 | 0.016 |
| **Sex, n (%)** | | | | | | |
| Male | 7603 (39.1) | 15345 (39.0) | 0.001 | 5490 (39.0) | 5421 (38.5) | 0.010 |
| Female | 11585 (59.6) | 23485 (59.7) | 0.003 | 8397 (59.7) | 8477 (60.3) | 0.012 |
| **Race, n (%)** | | | | | | |
| White | 14382 (73.9) | 30466 (77.5) | 0.082 | 10609 (75.4) | 10679 (75.9) | 0.012 |
| Black or African American | 2461 (12.7) | 4284 (10.9) | 0.055 | 1714 (12.2) | 1651 (11.7) | 0.014 |
| Asian | 817 (04.2) | 1616 (04.1) | 0.005 | 545 (03.9) | 558 (04.0) | 0.005 |
| Other race | 1696 (08.7) | 2754 (07.0) | 0.064 | 1130 (08.0) | 1122 (08.0) | 0.002 |
| **Social economic status, n (%)** | | | | | | |
| Housing/economic circumstances problems | 63 (00.3) | 102 (00.3) | 0.012 | 41 (00.3) | 38 (00.3) | 0.004 |
| **Lifestyles, n (%)** | | | | | | |
| Tobacco use | 444 (02.3) | 578 (01.5) | 0.060 | 268 (01.9) | 264 (01.9) | 0.002 |
| Nicotine dependence | 1513 (07.8) | 2075 (05.3) | **0.101** | 927 (06.6) | 917 (06.5) | 0.003 |
| Alcohol related disease | 559 (02.9) | 706 (01.8) | 0.071 | 348 (02.5) | 360 (02.6) | 0.005 |
| **Medical utilization, n (%)** | | | | | | |
| Office or other outpatient services | 17988 (92.5) | 32241 (82.0) | **0.319** | 12790 (90.9) | 12908 (91.8) | 0.030 |
| Emergency department services | 4074 (20.9) | 5705 (14.5) | **0.169** | 2527 (18.0) | 2462 (17.5) | 0.012 |
| Hospital inpatient services | 2210 (11.4) | 2588 (06.6) | **0.168** | 1268 (09.0) | 1238 (08.8) | 0.007 |
| Preventive medicine services | 1759 (09.0) | 4078 (10.4) | 0.045 | 1374 (09.8) | 1356 (09.6) | 0.004 |
| **Cancer site [b], n (%)** | | | | | | |
| Ill-defined, other secondary and unspecified sites | 6855 (35.2) | 3875 (09.9) | **0.638** | 3195 (22.7) | 3260 (23.2) | 0.011 |
| Breast | 5660 (29.1) | 8197 (20.8) | **0.192** | 3891 (27.7) | 3994 (28.4) | 0.016 |
| Lymphoid, hematopoietic and related tissue | 2870 (14.8) | 4655 (11.8) | 0.086 | 2066 (14.7) | 2138 (15.2) | 0.014 |

| | | | | | | |
|---|---|---|---|---|---|---|
| Male genital organs | 2271 (11.7) | 3444 (08.8) | 0.096 | 1596 (11.3) | 1649 (11.7) | 0.012 |
| Digestive organs | 2188 (11.2) | 2615 (06.6) | **0.162** | 1247 (08.9) | 1203 (08.6) | 0.011 |
| Melanoma and other malignant neoplasms of skin | 1843 (09.5) | 3598 (09.1) | 0.011 | 1413 (10.0) | 1536 (10.9) | 0.029 |
| Respiratory and intrathoracic organs | 1587 (08.2) | 1570 (04.0) | **0.175** | 879 (06.2) | 905 (06.4) | 0.008 |
| Urinary tract | 1112 (05.7) | 1824 (04.6) | 0.049 | 733 (05.2) | 738 (05.2) | 0.002 |
| Female genital organs | 1164 (06.0) | 1286 (03.3) | **0.129** | 652 (04.6) | 654 (04.6) | 0.001 |
| Mesothelial and soft tissue | 502 (02.6) | 402 (01.0) | **0.117** | 255 (01.8) | 247 (01.8) | 0.004 |
| Lip, oral cavity and pharynx | 419 (02.2) | 827 (02.1) | 0.004 | 303 (02.2) | 287 (02.0) | 0.008 |
| Eye, brain and other central nervous system parts | 355 (01.8) | 430 (01.1) | 0.061 | 215 (01.5) | 223 (01.6) | 0.005 |
| Thyroid and other endocrine glands | 308 (01.6) | 576 (01.5) | 0.010 | 213 (01.5) | 202 (01.4) | 0.006 |
| Malignant neuroendocrine tumors | 252 (01.3) | 291 (00.7) | 0.055 | 171 (01.2) | 159 (01.1) | 0.008 |
| Secondary neuroendocrine tumors | 146 (00.8) | 144 (00.4) | 0.052 | 94 (00.7) | 84 (00.6) | 0.009 |
| Bone and articular cartilage | 136 (00.7) | 120 (00.3) | 0.056 | 68 (00.5) | 63 (00.4) | 0.005 |
| **Comorbidities, n (%)** | | | | | | |
| Essential hypertension | 9874 (50.8) | 17995 (45.8) | 0.100 | 7027 (50.0) | 7122 (50.6) | 0.014 |
| Overweight and obesity | 2962 (15.2) | 4966 (12.6) | 0.075 | 2062 (14.7) | 2066 (14.7) | 0.001 |
| Hyperlipidemia, unspecified | 5979 (30.7) | 11101 (28.2) | 0.055 | 4321 (30.7) | 4364 (31.0) | 0.007 |
| Vitamin D deficiency | 2808 (14.4) | 5175 (13.2) | 0.037 | 2082 (14.8) | 2070 (14.7) | 0.002 |
| Diabetes mellitus | 3682 (18.9) | 7059 (17.9) | 0.025 | 2685 (19.1) | 2687 (19.1) | <0.001 |
| Asthma | 1801 (09.3) | 2925 (07.4) | 0.066 | 1265 (09.0) | 1291 (09.2) | 0.006 |
| Other chronic obstructive pulmonary disease | 1380 (07.1) | 2319 (05.9) | 0.049 | 950 (06.8) | 965 (06.9) | 0.004 |
| Diseases of liver | 2828 (14.5) | 3134 (08.0) | **0.209** | 1604 (11.4) | 1639 (11.7) | 0.008 |
| Disorders involving the immune mechanism | 8005 (41.2) | 9935 (25.3) | **0.342** | 4967 (35.3) | 4995 (35.5) | 0.004 |
| Depressive episode | 3135 (16.1) | 4741 (12.1) | **0.117** | 2102 (14.9) | 2093 (14.9) | 0.002 |
| Anxiety, stress-related, nonpsychotic mental disorders | 4248 (21.8) | 6098 (15.5) | **0.163** | 2752 (19.6) | 2760 (19.6) | 0.001 |
| Sleep disorders | 3886 (20.0) | 6291 (16.0) | **0.104** | 2663 (18.9) | 2673 (19.0) | 0.002 |
| **Laboratory, n (%)** | | | | | | |
| Leukocytes in Blood ($10^3$/uL) | | | | | | |
| < 4 | 4350 (22.4) | 3789 (09.6) | **0.353** | 2252 (16.0) | 2169 (15.4) | 0.016 |
| ≧11 | 3350 (17.2) | 3428 (08.7) | **0.255** | 1786 (12.7) | 1793 (12.7) | 0.001 |
| Lymphocytes/100 leukocytes in Blood (%) | | | | | | |
| < 20 | 7020 (36.1) | 7961 (20.2) | **0.358** | 4022 (28.6) | 4048 (28.8) | 0.004 |

| | | | | | | |
|---|---|---|---|---|---|---|
| ≧40 | 2916 (15.0) | 2825 (07.2) | **0.251** | 1527 (10.9) | 1533 (10.9) | 0.001 |
| Monocytes/100 leukocytes in Blood (%) | | | | | | |
| < 2 | 1689 (08.7) | 1152 (02.9) | **0.248** | 808 (05.7) | 775 (05.5) | 0.010 |
| ≧8 | 9662 (49.7) | 13476 (34.3) | **0.316** | 6058 (43.1) | 6024 (42.8) | 0.005 |
| Eosinophils/100 leukocytes in Blood (%) | | | | | | |
| < 1 | 6639 (34.1) | 7031 (17.9) | **0.377** | 3681 (26.2) | 3672 (26.1) | 0.001 |
| ≧4 | 5256 (27.0) | 5890 (15.0) | **0.299** | 3045 (21.6) | 3017 (21.4) | 0.005 |
| Basophils/100 leukocytes in Blood (%) | | | | | | |
| < 0.5 | 8151 (41.9) | 10068 (25.6) | **0.350** | 4907 (34.9) | 4914 (34.9) | 0.001 |
| ≧1 | 6557 (33.7) | 7809 (19.9) | **0.317** | 3853 (27.4) | 3825 (27.2) | 0.004 |
| Neutrophils/100 leukocytes in Blood (%) | | | | | | |
| < 40 | 1383 (07.1) | 1035 (02.6) | **0.209** | 677 (04.8) | 654 (04.6) | 0.008 |
| ≧60 | 7185 (36.9) | 9373 (23.8) | **0.288** | 4438 (31.5) | 4467 (31.8) | 0.004 |

Note: Bold font represents a standardized mean difference > 0.1.

If the patient is less or equal to 10, results show the count as 10.

PSM: Propensity score matching; SD: Standard deviation; SMD: Standardized mean difference;

a. Propensity score matching was performed on all listed characteristics.

b. Cancer site were identified using ICD-10-CM code, including lip, oral cavity, and pharynx (ICD-10 code C00-C14); digestive organs (C15-C26); respiratory and intrathoracic organs (C30-C39); bone and articular cartilage (C40-C41); melanoma and other malignant neoplasms (C43-C44); mesothelial and soft tissue (C45-C49); breast (C50); female genital organs (C51-C58); male genital organs (C60-C63); urinary tract (C64-C68); eye, brain, and other central nervous system components (C69-C72); thyroid and other endocrine glands (C73-C75); ill-defined, other secondary, and unspecified sites (C76-C80); lymphoid, hematopoietic, and related tissue (C81-C96); malignant neuroendocrine tumors (C7A), and secondary neuroendocrine tumors (C7B).

**Table S3.** Baseline characteristics of study subjects (18~64 years old) .

| Variables | Before PSM | | | After PSM [a] | | |
|---|---|---|---|---|---|---|
| | Time lag within 3 months (n=7,932) | Time lag at least 3 months (n=13,819) | SMD | Time lag within 3 months (n=5,437) | Time lag at least 3 months (n=5,437) | SMD |
| **Age at index, y** | | | | | | |
| Mean ± SD | 53.2 ± 9.7 | 53.3 ± 10.0 | 0.008 | 53.6 ± 9.6 | 53.6 ± 9.7 | 0.000 |
| **Sex, n (%)** | | | | | | |
| Male | 2641 (33.3) | 4822 (34.9) | 0.034 | 1833 (33.7) | 1798 (33.1) | 0.014 |
| Female | 5172 (65.2) | 8805 (63.7) | 0.031 | 3517 (64.7) | 3543 (65.2) | 0.010 |
| **Race, n (%)** | | | | | | |
| White | 5447 (68.7) | 9911 (71.7) | 0.067 | 3798 (69.9) | 3734 (68.7) | 0.026 |
| Black or African American | 1167 (14.7) | 1802 (13.0) | 0.048 | 779 (14.3) | 810 (14.9) | 0.016 |
| Asian | 361 (4.6) | 663 (4.8) | 0.012 | 243 (4.5) | 248 (4.6) | 0.004 |
| Other Race | 908 (11.4) | 1345 (9.7) | 0.056 | 582 (10.7) | 607 (11.2) | 0.015 |
| **Social economic status, n (%)** | | | | | | |
| Housing/economic circumstances problems | 36 (0.5) | 64 (0.5) | 0.001 | 20 (0.4) | 22 (0.4) | 0.006 |
| **Lifestyles, n (%)** | | | | | | |
| Tobacco use | 241 (3.0) | 264 (1.9) | 0.073 | 142 (2.6) | 132 (2.4) | 0.012 |
| Nicotine dependence | 770 (9.7) | 914 (6.6) | **0.113** | 464 (8.5) | 441 (8.1) | 0.015 |
| Alcohol related disorders | 272 (3.4) | 275 (2.0) | 0.089 | 155 (2.9) | 162 (3.0) | 0.008 |
| **Medical utilization, n (%)** | | | | | | |
| Office or Other Outpatient Services | 7325 (92.3) | 11081 (80.2) | **0.359** | 4906 (90.2) | 4918 (90.5) | 0.007 |
| Emergency Department Services | 1692 (21.3) | 1974 (14.3) | **0.185** | 960 (17.7) | 973 (17.9) | 0.006 |
| Preventive Medicine Services | 1104 (13.9) | 2387 (17.3) | 0.093 | 851 (15.7) | 793 (14.6) | 0.030 |

| | | | | | | |
|---|---|---|---|---|---|---|
| Hospital Inpatient and Observation Care Services | 927 (11.7) | 826 (6.0) | 0.202 | 495 (9.1) | 479 (8.8) | 0.010 |
| **Cancer site [b], n (%)** | | | | | | |
| Ill-defined, other secondary and unspecified sites | 2975 (37.5) | 1517 (11.0) | **0.651** | 1283 (23.6) | 1329 (24.4) | 0.020 |
| Breast | 2640 (33.3) | 2951 (21.4) | **0.270** | 1659 (30.5) | 1693 (31.1) | 0.014 |
| Lymphoid, hematopoietic and related tissue | 1212 (15.3) | 1977 (14.3) | 0.027 | 863 (15.9) | 877 (16.1) | 0.007 |
| Digestive organs | 936 (11.8) | 905 (6.5) | **0.183** | 484 (8.9) | 495 (9.1) | 0.007 |
| Melanoma and other malignant neoplasms of skin | 565 (7.1) | 887 (6.4) | 0.028 | 389 (7.2) | 413 (7.6) | 0.017 |
| Male genital organs | 453 (5.7) | 652 (4.7) | 0.045 | 318 (5.8) | 321 (5.9) | 0.002 |
| Respiratory and intrathoracic organs | 554 (7.0) | 413 (3.0) | **0.184** | 285 (5.2) | 280 (5.2) | 0.004 |
| Female genital organs | 508 (6.4) | 512 (3.7) | **0.123** | 282 (5.2) | 296 (5.4) | 0.011 |
| Urinary tract | 315 (4.0) | 403 (2.9) | 0.058 | 196 (3.6) | 181 (3.3) | 0.015 |
| Eye, brain and other central nervous system parts | 220 (2.8) | 294 (2.1) | 0.042 | 143 (2.6) | 130 (2.4) | 0.015 |
| Lip, oral cavity and pharynx | 182 (2.3) | 362 (2.6) | 0.021 | 125 (2.3) | 129 (2.4) | 0.005 |
| Mesothelial and soft tissue | 255 (3.2) | 182 (1.3) | **0.128** | 124 (2.3) | 123 (2.3) | 0.001 |
| Thyroid and other endocrine glands | 157 (2.0) | 302 (2.2) | 0.014 | 108 (2.0) | 119 (2.2) | 0.014 |
| Malignant neuroendocrine tumors | 98 (1.2) | 131 (0.9) | 0.028 | 59 (1.1) | 63 (1.2) | 0.007 |
| Secondary neuroendocrine tumors | 64 (0.8) | 65 (0.5) | 0.042 | 36 (0.7) | 34 (0.6) | 0.005 |
| Bone and articular cartilage | 80 (1.0) | 66 (0.5) | 0.062 | 30 (0.6) | 37 (0.7) | 0.016 |
| **Comorbidities, n (%)** | | | | | | |
| Essential (primary) hypertension | 2902 (36.6) | 4299 (31.1) | **0.116** | 1938 (35.6) | 1920 (35.3) | 0.007 |
| Disorders involving the immune mechanism | 3274 (41.3) | 3367 (24.4) | **0.366** | 1878 (34.5) | 1879 (34.6) | 0.000 |
| Anxiety, stress-related, nonpsychotic mental disorders | 2159 (27.2) | 2634 (19.1) | **0.194** | 1288 (23.7) | 1292 (23.8) | 0.002 |
| Hyperlipidemia, unspecified | 1472 (18.6) | 2375 (17.2) | 0.036 | 1001 (18.4) | 982 (18.1) | 0.009 |
| Sleep disorders | 1546 (19.5) | 2084 (15.1) | **0.117** | 965 (17.7) | 978 (18.0) | 0.006 |
| Depressive episode | 1485 (18.7) | 1916 (13.9) | **0.132** | 908 (16.7) | 933 (17.2) | 0.012 |
| Overweight and obesity | 1347 (17.0) | 1881 (13.6) | 0.094 | 895 (16.5) | 867 (15.9) | 0.014 |
| Diabetes mellitus | 1144 (14.4) | 1906 (13.8) | 0.018 | 792 (14.6) | 789 (14.5) | 0.002 |

| | | | | | | |
|---|---|---|---|---|---|---|
| Vitamin D deficiency | 1063 (13.4) | 1834 (13.3) | 0.004 | 771 (14.2) | 788 (14.5) | 0.009 |
| Diseases of liver | 1260 (15.9) | 1222 (8.8) | **0.215** | 641 (11.8) | 686 (12.6) | 0.025 |
| Asthma | 824 (10.4) | 1148 (8.3) | 0.072 | 543 (10.0) | 549 (10.1) | 0.004 |
| Other chronic obstructive pulmonary disease | 334 (4.2) | 457 (3.3) | 0.048 | 213 (3.9) | 199 (3.7) | 0.013 |
| **Laboratory, n (%)** | | | | | | |
| Leukocytes in Blood ($10^3$/uL) | | | | | | |
| < 4 | 1958 (24.7) | 1579 (11.4) | **0.350** | 972 (17.9) | 952 (17.5) | 0.010 |
| ≧11 | 1504 (19.0) | 1248 (9.0) | **0.289** | 754 (13.9) | 715 (13.2) | 0.021 |
| Lymphocytes/100 leukocytes in Blood (%) | | | | | | |
| < 20 | 2713 (34.2) | 2402 (17.4) | **0.392** | 1401 (25.8) | 1391 (25.6) | 0.004 |
| ≧40 | 1400 (17.7) | 1305 (9.4) | **0.242** | 740 (13.6) | 728 (13.4) | 0.006 |
| Monocytes/100 leukocytes in Blood (%) | | | | | | |
| < 2 | 807 (10.2) | 505 (3.7) | **0.259** | 391 (7.2) | 361 (6.6) | 0.022 |
| ≧8 | 3813 (48.1) | 4531 (32.8) | **0.315** | 2245 (41.3) | 2246 (41.3) | 0.000 |
| Eosinophils/100 leukocytes in Blood (%) | | | | | | |
| < 1 | 2884 (36.4) | 2710 (19.6) | **0.380** | 1529 (28.1) | 1517 (27.9) | 0.005 |
| ≧4 | 2142 (27.0) | 2085 (15.1) | **0.296** | 1173 (21.6) | 1146 (21.1) | 0.012 |
| Basophils/100 leukocytes in Blood (%) | | | | | | |
| < 0.5 | 3378 (42.6) | 3736 (27.0) | **0.331** | 1934 (35.6) | 1921 (35.3) | 0.005 |
| ≧1 | 2736 (34.5) | 2851 (20.6) | **0.314** | 1531 (28.2) | 1533 (28.2) | 0.001 |
| Neutrophils/100 leukocytes in Blood (%) | | | | | | |
| < 40 | 625 (7.9) | 476 (3.4) | **0.193** | 302 (5.6) | 303 (5.6) | 0.001 |
| ≧60 | 2759 (34.8) | 2968 (21.5) | **0.299** | 1575 (29.0) | 1571 (28.9) | 0.002 |

Note: Bold font represents a standardized mean difference > 0.1.
If the patient is less or equal to 10, results show the count as 10.
PSM: Propensity score matching; SD: Standard deviation; SMD: Standardized mean difference;

a. Propensity score matching was performed on all listed characteristics.
b. Cancer site were identified using ICD-10-CM code, including lip, oral cavity, and pharynx (ICD-10 code C00-C14); digestive organs (C15-C26); respiratory and

intrathoracic organs (C30-C39); bone and articular cartilage (C40-C41); melanoma and other malignant neoplasms (C43-C44); mesothelial and soft tissue (C45-C49); breast (C50); female genital organs (C51-C58); male genital organs (C60-C63); urinary tract (C64-C68); eye, brain, and other central nervous system components (C69-C72); thyroid and other endocrine glands (C73-C75); ill-defined, other secondary, and unspecified sites (C76-C80); lymphoid, hematopoietic, and related tissue (C81-C96); malignant neuroendocrine tumors (C7A), and secondary neuroendocrine tumors (C7B).

**Table S4.** Baseline characteristics of study subjects (≧ 65 years old) .

| | Before PSM | | | After PSM | | |
|---|---|---|---|---|---|---|
| | Time lag within 3 months (n=11,533) | Time lag at least 3 months (n=25,545) | SMD | Time lag within 3 months (n=8,616) | Time lag at least 3 months (n=8,616) | SMD |
| **Age at index, y** | | | | | | |
| Mean ± SD | 73.6 ± 5.8 | 74.2 ± 6.0 | **0.108** | 73.8 ± 5.9 | 73.8 ± 5.9 | 0.008 |
| **Sex, n (%)** | | | | | | |
| Male | 4946 (42.9) | 10517 (41.2) | 0.035 | 3615 (42.0) | 3618 (42.0) | 0.001 |
| Female | 6402 (55.5) | 14640 (57.3) | 0.036 | 4869 (56.5) | 4864 (56.5) | 0.001 |
| **Race, n (%)** | | | | | | |
| White | 8914 (77.3) | 20521 (80.3) | 0.074 | 6769 (78.6) | 6808 (79.0) | 0.011 |
| Black or African American | 1288 (11.2) | 2472 (9.7) | 0.049 | 924 (10.7) | 911 (10.6) | 0.005 |
| Asian | 456 (4.0) | 953 (3.7) | 0.012 | 303 (3.5) | 301 (3.5) | 0.001 |
| Other Race | 828 (7.2) | 1487 (5.8) | 0.055 | 585 (6.8) | 568 (6.6) | 0.008 |
| **Social economic status, n (%)** | | | | | | |
| Housing/economic circumstances problems | 27 (0.2) | 38 (0.1) | 0.020 | 18 (0.2) | 14 (0.2) | 0.011 |
| **Lifestyles, n (%)** | | | | | | |
| Tobacco use | 203 (1.8) | 314 (1.2) | 0.044 | 131 (1.5) | 115 (1.3) | 0.016 |
| Nicotine dependence | 743 (6.4) | 1161 (4.5) | 0.083 | 469 (5.4) | 457 (5.3) | 0.006 |
| Alcohol related disorders | 287 (2.5) | 431 (1.7) | 0.056 | 197 (2.3) | 195 (2.3) | 0.002 |
| **Medical utilization, n (%)** | | | | | | |
| Office or Other Outpatient Services | 10676 (92.6) | 21188 (82.9) | **0.297** | 7867 (91.3) | 7945 (92.2) | 0.033 |
| Emergency Department Services | 2388 (20.7) | 3738 (14.6) | **0.160** | 1539 (17.9) | 1549 (18.0) | 0.003 |
| Hospital Inpatient and Observation Care Services | 1286 (11.2) | 1767 (6.9) | **0.148** | 778 (9.0) | 764 (8.9) | 0.006 |

| | | | | | | |
|---|---|---|---|---|---|---|
| Preventive Medicine Services | 655 (5.7) | 1693 (6.6) | 0.039 | 520 (6.0) | 503 (5.8) | 0.008 |
| **Cancer site [b], n (%)** | | | | | | |
| Breast | 3022 (26.2) | 5247 (20.5) | **0.134** | 2238 (26.0) | 2307 (26.8) | 0.018 |
| Ill-defined, other secondary and unspecified sites | 3883 (33.7) | 2359 (9.2) | **0.623** | 1916 (22.2) | 1932 (22.4) | 0.004 |
| Lymphoid, hematopoietic and related tissue | 1662 (14.4) | 2679 (10.5) | **0.119** | 1200 (13.9) | 1191 (13.8) | 0.003 |
| Digestive organs | 1252 (10.9) | 1711 (6.7) | **0.147** | 724 (8.4) | 765 (8.9) | 0.017 |
| Melanoma and other malignant neoplasms of skin | 1280 (11.1) | 2715 (10.6) | 0.015 | 1007 (11.7) | 1048 (12.2) | 0.015 |
| Male genital organs | 1818 (15.8) | 2797 (10.9) | **0.142** | 1274 (14.8) | 1321 (15.3) | 0.015 |
| Respiratory and intrathoracic organs | 1036 (9.0) | 1158 (4.5) | **0.178** | 612 (7.1) | 601 (7.0) | 0.005 |
| Female genital organs | 658 (5.7) | 775 (3.0) | **0.131** | 394 (4.6) | 376 (4.4) | 0.010 |
| Urinary tract | 798 (6.9) | 1422 (5.6) | 0.056 | 557 (6.5) | 523 (6.1) | 0.016 |
| Eye, brain and other central nervous system parts | 135 (1.2) | 136 (0.5) | 0.069 | 72 (0.8) | 66 (0.8) | 0.008 |
| Lip, oral cavity and pharynx | 237 (2.1) | 465 (1.8) | 0.017 | 155 (1.8) | 158 (1.8) | 0.003 |
| Mesothelial and soft tissue | 249 (2.2) | 220 (0.9) | **0.107** | 124 (1.4) | 124 (1.4) | 0.000 |
| Thyroid and other endocrine glands | 151 (1.3) | 274 (1.1) | 0.022 | 107 (1.2) | 111 (1.3) | 0.004 |
| Malignant neuroendocrine tumors | 154 (1.3) | 160 (0.6) | 0.072 | 93 (1.1) | 95 (1.1) | 0.002 |
| Secondary neuroendocrine tumors | 82 (0.7) | 79 (0.3) | 0.056 | 49 (0.6) | 50 (0.6) | 0.002 |
| Bone and articular cartilage | 56 (0.5) | 54 (0.2) | 0.047 | 34 (0.4) | 29 (0.3) | 0.010 |
| **Comorbidities, n (%)** | | | | | | |
| Essential (primary) hypertension | 6980 (60.5) | 13720 (53.7) | **0.138** | 5081 (59.0) | 5140 (59.7) | 0.014 |
| Disorders involving the immune mechanism | 4738 (41.1) | 6578 (25.8) | **0.329** | 3068 (35.6) | 3068 (35.6) | 0.000 |
| Anxiety, stress-related, nonpsychotic mental disorders | 2090 (18.1) | 3470 (13.6) | **0.124** | 1432 (16.6) | 1412 (16.4) | 0.006 |
| Hyperlipidemia, unspecified | 4508 (39.1) | 8731 (34.2) | **0.102** | 3255 (37.8) | 3324 (38.6) | 0.016 |
| Sleep disorders | 2342 (20.3) | 4210 (16.5) | 0.099 | 1681 (19.5) | 1699 (19.7) | 0.005 |
| Depressive episode | 1651 (14.3) | 2829 (11.1) | 0.097 | 1156 (13.4) | 1119 (13.0) | 0.013 |
| Overweight and obesity | 1616 (14.0) | 3086 (12.1) | 0.057 | 1179 (13.7) | 1165 (13.5) | 0.005 |
| Diabetes mellitus | 2538 (22.0) | 5159 (20.2) | 0.044 | 1871 (21.7) | 1886 (21.9) | 0.004 |

| | | | | | | |
|---|---|---|---|---|---|---|
| Vitamin D deficiency | 1746 (15.1) | 3344 (13.1) | 0.059 | 1294 (15.0) | 1321 (15.3) | 0.009 |
| Diseases of liver | 1568 (13.6) | 1913 (7.5) | **0.200** | 917 (10.6) | 970 (11.3) | 0.020 |
| Asthma | 977 (8.5) | 1778 (7.0) | 0.057 | 727 (8.4) | 726 (8.4) | 0.000 |
| Other chronic obstructive pulmonary disease | 1048 (9.1) | 1863 (7.3) | 0.065 | 759 (8.8) | 702 (8.1) | 0.024 |
| **Laboratory, n (%)** | | | | | | |
| Leukocytes in Blood ($10^3$/uL) | | | | | | |
| < 4 | 2396 (20.8) | 2212 (8.7) | **0.347** | 1272 (14.8) | 1218 (14.1) | 0.018 |
| ≧11 | 1851 (16.1) | 2183 (8.5) | **0.230** | 1052 (12.2) | 1038 (12.0) | 0.005 |
| Lymphocytes/100 leukocytes in Blood (%) | | | | | | |
| < 20 | 4315 (37.4) | 5571 (21.8) | **0.347** | 2644 (30.7) | 2599 (30.2) | 0.011 |
| ≧40 | 1519 (13.2) | 1521 (6.0) | **0.247** | 832 (9.7) | 790 (9.2) | 0.017 |
| Monocytes/100 leukocytes in Blood (%) | | | | | | |
| < 2 | 884 (7.7) | 649 (2.5) | **0.234** | 423 (4.9) | 390 (4.5) | 0.018 |
| ≧8 | 5856 (50.8) | 8958 (35.1) | **0.321** | 3822 (44.4) | 3810 (44.2) | 0.003 |
| Eosinophils/100 leukocytes in Blood (%) | | | | | | |
| < 1 | 3765 (32.6) | 4327 (16.9) | **0.370** | 2198 (25.5) | 2154 (25.0) | 0.012 |
| ≧4 | 3116 (27.0) | 3808 (14.9) | **0.301** | 1879 (21.8) | 1826 (21.2) | 0.015 |
| Basophils/100 leukocytes in Blood (%) | | | | | | |
| < 0.5 | 4782 (41.5) | 6340 (24.8) | **0.359** | 2990 (34.7) | 2974 (34.5) | 0.004 |
| ≧1 | 3829 (33.2) | 4964 (19.4) | **0.317** | 2350 (27.3) | 2296 (26.6) | 0.014 |
| Neutrophils/100 leukocytes in Blood (%) | | | | | | |
| < 40 | 760 (6.6) | 560 (2.2) | **0.216** | 359 (4.2) | 342 (4.0) | 0.010 |
| ≧60 | 4433 (38.4) | 6418 (25.1) | **0.289** | 2858 (33.2) | 2848 (33.1) | 0.002 |

Note: Bold font represents a standardized mean difference > 0.1.
If the patient is less or equal to 10, results show the count as 10.
PSM: Propensity score matching; SD: Standard deviation; SMD: Standardized mean difference;

a. Propensity score matching was performed on all listed characteristics.
b. Cancer site were identified using ICD-10-CM code, including lip, oral cavity, and pharynx (ICD-10 code C00-C14); digestive organs (C15-C26); respiratory and

intrathoracic organs (C30-C39); bone and articular cartilage (C40-C41); melanoma and other malignant neoplasms (C43-C44); mesothelial and soft tissue (C45-C49); breast (C50); female genital organs (C51-C58); male genital organs (C60-C63); urinary tract (C64-C68); eye, brain, and other central nervous system components (C69-C72); thyroid and other endocrine glands (C73-C75); ill-defined, other secondary, and unspecified sites (C76-C80); lymphoid, hematopoietic, and related tissue (C81-C96); malignant neuroendocrine tumors (C7A), and secondary neuroendocrine tumors (C7B).

**Table S5.** Risk of outcomes (1 day to 30 days)_4 models**.**

| **Outcomes** (Time lag within 3 months vs. time lag at least 3 months) | **Hazard ratio (95% CI)** | | | |
|---|---|---|---|---|
| | **Model 1[a]** | **Model 2[b]** | **Model 3[c]** | **Model 4[d]** |
| **Infections** | | | | |
| COVID-19 infection | **1.396 (1.100-1.770)** | **1.454 (1.068-1.980)** | **1.532 (1.112-2.109)** | **1.428 (1.035-1.970)** |
| Pneumonia | **1.657 (1.260-2.181)** | 1.252 (0.878-1.784) | 1.095 (0.776-1.545) | 1.178 (0.808-1.717) |
| Skin infection | 1.140 (0.861-1.511) | 1.149 (0.811-1.629) | 1.081 (0.761-1.536) | 0.961 (0.657-1.406) |
| Intra-abdominal infection | 1.611 (0.636-4.082) | 1.663 (0.397-6.957) | 0.999 (0.202-4.948) | 0.249 (0.028-2.231) |
| Urinary tract infection | **1.632 (1.311-2.032)** | **1.436 (1.079-1.910)** | **1.321 (1.000-1.745)** | **1.477 (1.083-2.014)** |
| Severe infection (Sepsis) | **2.582 (1.803-3.698)** | **1.889 (1.195-2.985)** | **1.790 (1.137-2.820)** | **1.854 (1.091-3.152)** |
| **Medical utilization** | | | | |
| Hospital inpatient services | **2.436 (2.086-2.844)** | **1.988 (1.616-2.444)** | **1.855 (1.512-2.274)** | **1.692 (1.354-2.115)** |
| Critical care services | **2.342 (1.633-3.358)** | **2.314 (1.404-3.815)** | **1.783 (1.123-2.832)** | 1.343 (0.831-2.172) |
| **All-cause mortality** | | | | |
| Deceased | **2.010 (1.341-3.011)** | **1.804 (1.059-3.075)** | 1.711 (0.999-2.930) | 1.995 (0.998-3.989) |

Note:
CI: Confidence interval. COVID-19: Coronavirus disease 2019.
a. Before matching, crude hazard ratio.
b. Propensity score matching was performed on age at index, sex, race, and cancer site.
c. Propensity score matching was performed on age at index, sex, race, cancer site, social economic status, lifestyles, and medical utilization.
d. Propensity score matching was performed on all listed characteristics.
*Proportionality <0.001

**Figure S1**. Kaplan-Meier curve of urinary tract infection.

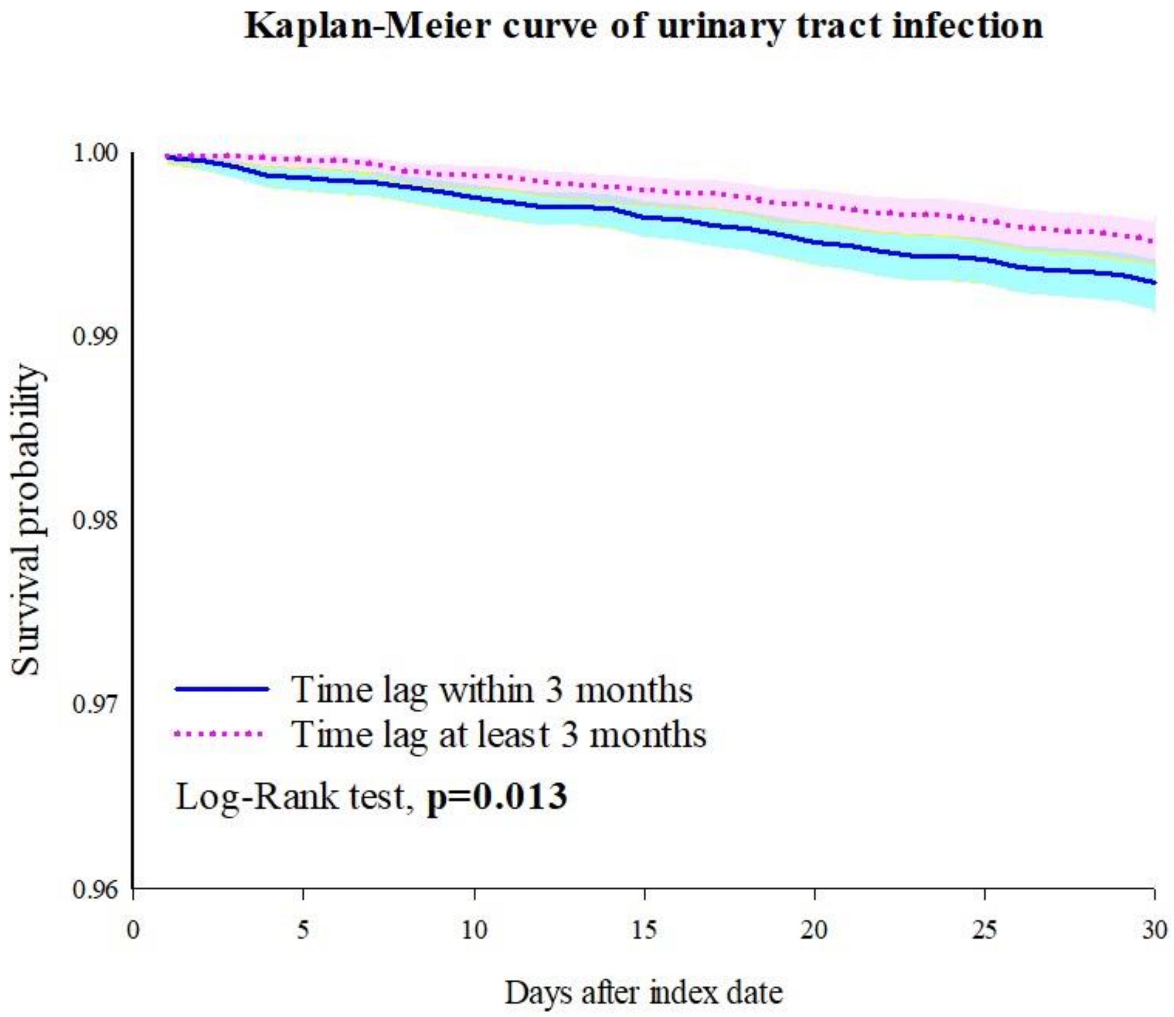

**Figure S2.** Kaplan-Meier curve of severe infection (Sepsis) .

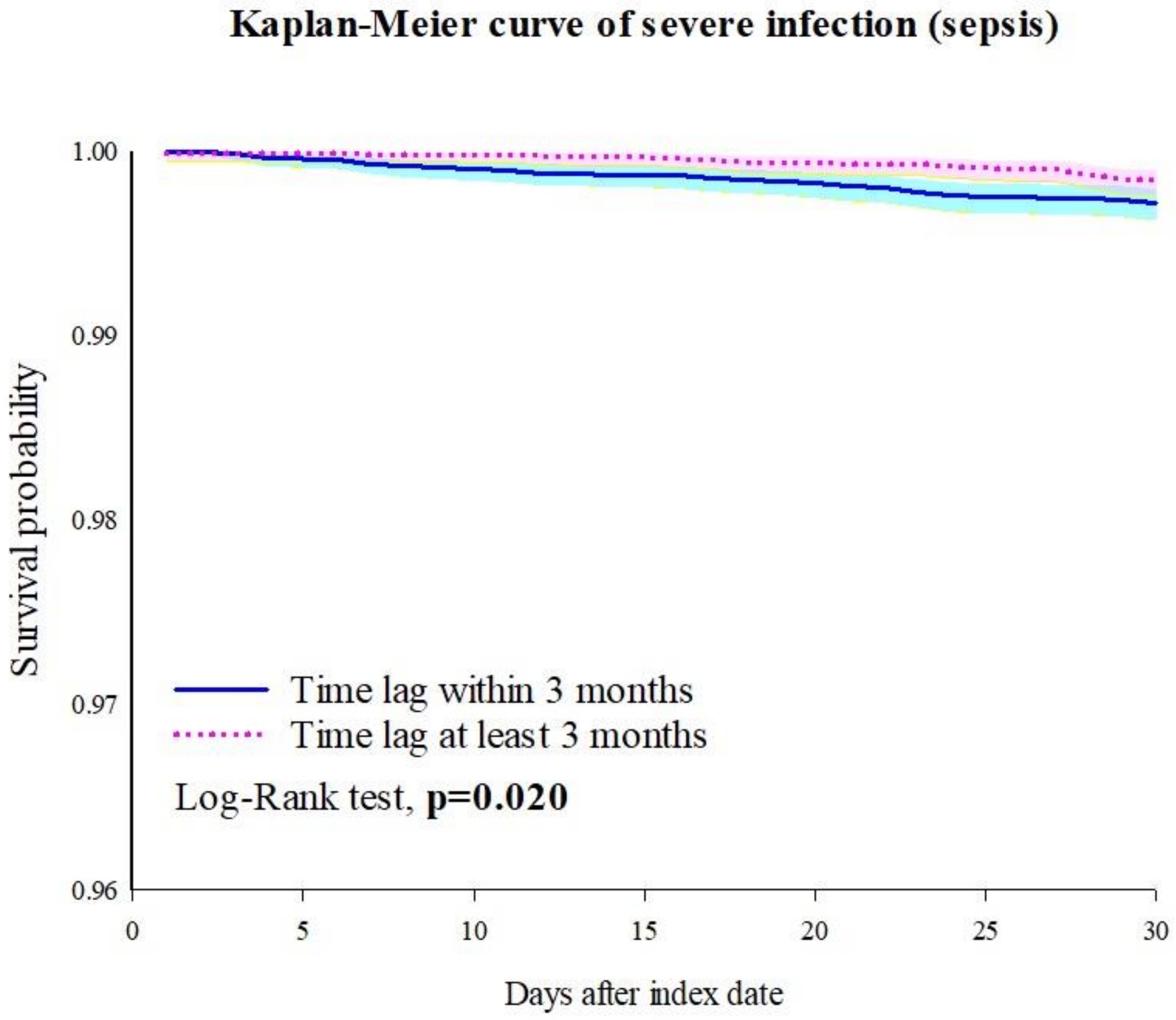

**Figure S3.** Kaplan-Meier curve of hospitalization.

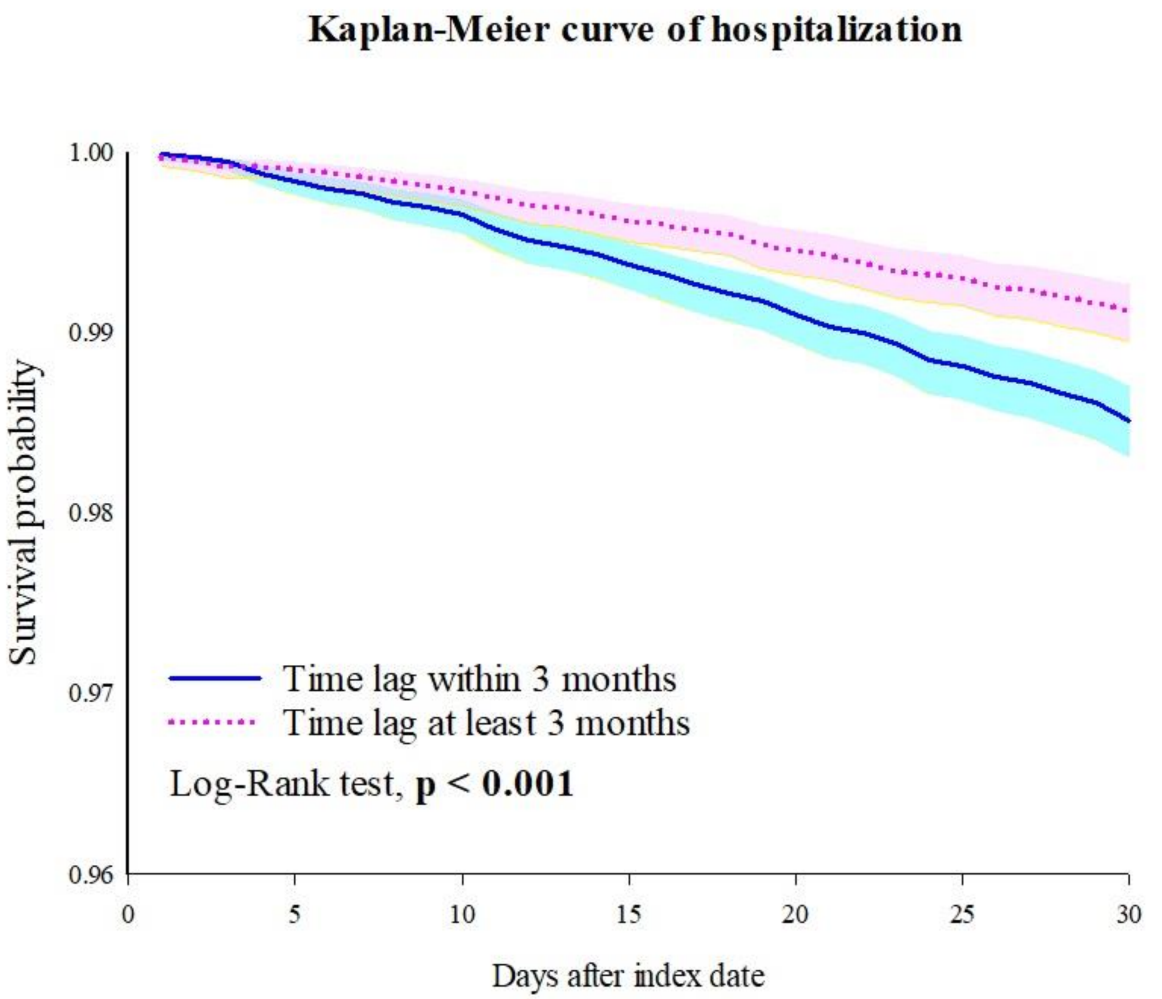

**Table S6.** Risk of outcomes (1 day to 30 days)_stratified by cancer type**.**

| **Outcomes**<br>(Time lag within 3 months vs. time lag at least 3 months) | **Adjusted hazard ratio (95% CI)[a]** | |
|---|---|---|
| | **Solid organ cancers[b]**<br>(n=12406 vs. 12406) | **Lymphoid, hematopoietic and related tissue cancers[c]**<br>(n=2722 vs. 2722) |
| **Infections** | | |
| COVID-19 infection | 0.952 (0.672-1.348) | 1.502 (0.798-2.827) |
| Pneumonia | 1.199 (0.788-1.823) | 0.808 (0.426-1.531) |
| Skin infection | 1.156 (0.749-1.784) | 0.713 (0.317-1.605) |
| Intra-abdominal infection | 0.749 (0.168-3.344) | 0.500 (0.045-5.517) |
| Urinary tract infection | 1.299 (0.928-1.819) | 2.129 (0.919-4.933) |
| Severe infection (Sepsis) | 1.497 (0.850-2.637) | 1.100 (0.467-2.589) |
| **Medical utilization** | | |
| Hospital inpatient services | **1.784 (1.392-2.288)** | 1.024 (0.672-1.558) |
| Critical care services | **2.197 (1.193-4.045)** | 0.999 (0.289-3.450) |
| **All-cause mortality** | | |
| Deceased | 1.263 (0.642-2.486) | 0.833 (0.254-2.729) |

Note:
CI: Confidence interval. NA: Not available. COVID-19: Coronavirus disease 2019.
a. Propensity score matching was performed on all listed characteristics.
b. Defined by ICD 10 code:C00-C75.
c. Defined by ICD 10 code:C81-C96.
* Proportionality <0.001.

**Table S7.** Risk of outcomes (1 day to 30 days)_stratified by vaccine.

| **Outcomes**<br>(Time lag within 3 months vs. time lag at least 3 months) | **Adjusted hazard ratio (95% CI)[a]** | |
|---|---|---|
| | **BNT162b2**<br>(n=10551 vs. 10551) | **mRNA-1273**<br>(n=3537 vs. 3537) |
| **Infections** | | |
| COVID-19 infection | 1.229 (0.853-1.773) | 1.334 (0.683-2.605) |
| Pneumonia | 0.839 (0.542-1.299) | 0.999 (0.463-2.154) |
| Skin infection | 0.912 (0.565-1.473) | 1.076 (0.506-2.290) |
| Intra-abdominal infection | 0.249 (0.028-2.229) | NA |
| Urinary tract infection | **1.717 (1.157-2.548)** | 1.319 (0.758-2.295) |
| Severe infection (Sepsis) | 1.262 (0.691-2.303) | 0.922 (0.421-2.021) |
| **Medical utilization** | | |
| Hospital inpatient services | **1.862 (1.416-2.447)** | 1.335 (0.881-2.021) |
| Critical care services | 1.597 (0.838-3.045) | 0.733 (0.336-1.595) |
| **All-cause mortality** | | |
| Deceased | 1.426 (0.720-2.822) | 0.999 (0.289-3.452) |

Note:
CI: Confidence interval. COVID-19: Coronavirus disease 2019. NA: Not available.
a. Propensity score matching was performed on all listed characteristics.
*Proportionality <0.001.

**Table S8.** Risk of outcomes (1 day to 30 days)_stratified by sex.

| **Outcomes**<br>(Time lag within 3 months vs. time lag at least 3 months) | **Adjusted hazard ratio (95% CI)** [a] | |
|---|---|---|
| | **Male**<br>(n=5357 vs. 5357) | **Female**<br>(n=8540 vs. 8540) |
| **Infections** | | |
| COVID-19 infection | 1.161 (0.718-1.877) | 1.351 (0.867-2.105) |
| Pneumonia | 1.068 (0.643-1.771) | 1.260 (0.690-2.300) |
| Skin infection | 0.999 (0.553-1.803) | 1.190 (0.707-2.004) |
| Intra-abdominal infection | 0.499 (0.045-5.506) | 0.996 (0.140-7.069) |
| Urinary tract infection | 1.037 (0.605-1.778) | 1.403 (0.965-2.039) |
| Severe infection (Sepsis) | 0.999 (0.510-1.957) | 1.885 (0.840-4.228) |
| **Medical utilization** | | |
| Hospital inpatient services | 1.277 (0.923-1.767) | **1.819 (1.331-2.486)** |
| Critical care services | 1.500 (0.763-2.949) | 1.282 (0.638-2.578) |
| **All-cause mortality** | | |
| Deceased | 1.075 (0.505-2.287) | 2.394 (0.843-6.795) |

Note:
CI: Confidence interval. COVID-19: Coronavirus disease 2019. NA: Not available.
a. Propensity score matching was performed on all listed characteristics.
*Proportionality <0.001.

**Table S9.** Risk of outcomes (1 day to 30 days)_stratified by age at index**.**

| **Outcomes**<br>(Time lag within 3 months vs. time lag at least 3 months) | **Adjusted hazard ratio (95% CI)** [a] | |
|---|---|---|
| | **18-64y**<br>(n=5437 vs. 5437) | **≧ 65y**<br>(n=8616 vs. 8616) |
| **Infections** | | |
| COVID-19 infection | **1.607 (1.002-2.575)** | 1.228 (0.786-1.918) |
| Pneumonia | 1.305 (0.634-2.688) | 0.919 (0.581-1.455) |
| Skin infection | 1.060 (0.535-2.097) | 1.358 (0.810-2.276) |
| Intra-abdominal infection | 2.994 (0.311-28.78) | NA |
| Urinary tract infection | **2.697 (1.305-5.571)** | 1.115 (0.787-1.580) |
| Severe infection (Sepsis) | 1.796 (0.602-5.360) | 1.685 (0.908-3.126) |
| **Medical utilization** | | |
| Hospital inpatient services | **1.800 (1.191-2.722)** | **1.812 (1.376-2.384)** |
| Critical care services | 1.746 (0.733-4.163) | 1.730 (0.916-3.266) |
| **All-cause mortality** | | |
| Deceased | 6.985 (0.859-56.77) | 0.577 (0.275-1.213) |

Note:
CI: Confidence interval. COVID-19: Coronavirus disease 2019. NA: Not available.
a. Propensity score matching was performed on all listed characteristics.
*Proportionality <0.001.

**Table S10.** Risk of outcomes (1 days to 30 days)_deal with competing risk (deceased) **.**

| Outcomes | Patients with outcome | | Adjusted hazard ratio (95%CI) [a] |
|---|---|---|---|
| | Time lag within 3 months | Time lag at least 3 months | |
| **Infections** | | | |
| COVID-19 infection | 103 | 83 | 1.239 (0.928-1.654) |
| Pneumonia | 81 | 64 | 1.263 (0.910-1.753) |
| Skin infection | 66 | 63 | 1.045 (0.740-1.476) |
| Intra-abdominal infection | 25 | 21 | 1.187 (0.664-2.120) |
| Urinary tract infection | 112 | 88 | 1.271 (0.961-1.680) |
| Severe infection (Sepsis) | 56 | 36 | **1.552 (1.021-2.359)** |
| **Medical utilization** | | | |
| Hospital inpatient services | 219 | 135 | **1.622 (1.309-2.010)** |
| Critical care services | 61 | 41 | **1.485 (1.000-2.206)** |
| **All-cause mortality** | | | |
| Deceased | 22 | 17 | 1.290 (0.685-2.429) |

Note:
CI: Confidence interval. COVID-19: Coronavirus disease 2019. NA: Not available.
If the patient is less or equal to 10, results show the count as 10.
a. Propensity score matching was performed on all listed characteristics.
* Proportionality <0.001.
